# Ten-year experience with pharmacogenetic testing for *DPYD* in a national cancer center in Italy: Lessons learned on the path to implementation

**DOI:** 10.3389/fphar.2023.1199462

**Published:** 2023-05-15

**Authors:** A. Bignucolo, E. De Mattia, R. Roncato, E. Peruzzi, L. Scarabel, M. D’Andrea, F. Sartor, G. Toffoli, E. Cecchin

**Affiliations:** Experimental and Clinical Pharmacology, Centro di Riferimento Oncologico di Aviano (CRO) IRCCS, Aviano, Italy

**Keywords:** pharmacogenetics, *DPYD*, implementation, genotyping, phenotyping, CDSS

## Abstract

**Background:** Awareness about the importance of implementing *DPYD* pharmacogenetics in clinical practice to prevent severe side effects related to the use of fluoropyrimidines has been raised over the years. Since 2012 at the National Cancer Institute, CRO-Aviano (Italy), a diagnostic *DPYD* genotyping service was set up.

**Purpose:** This study aims to describe the evolution of *DPYD* diagnostic activity at our center over the last 10 years as a case example of a successful introduction of pharmacogenetic testing in clinical practice.

**Methods:** Data related to the diagnostic activity of in–and out-patients referred to our service between January 2012 and December 2022 were retrieved from the hospital database.

**Results:** *DPYD* diagnostic activity at our center has greatly evolved over the years, shifting gradually from a post-toxicity to a pre-treatment approach. Development of pharmacogenetic guidelines by national and international consortia, genotyping, and IT technology evolution have impacted *DPYD* testing uptake in the clinics. Our participation in a large prospective implementation study (Ubiquitous Pharmacogenomics) increased health practitioners’ and patients’ awareness of pharmacogenetic matters and provided additional standardized infrastructures for genotyping and reporting. Nationwide test reimbursement together with recommendations by regulatory agencies in Europe and Italy in 2020 definitely changed the clinical practice guidelines of fluoropyrimidines prescription. A dramatic increase in the number of pre-treatment *DPYD* genotyping and in the coverage of new fluoropyrimidine prescriptions was noticed by the last year of observation (2022).

**Conclusion:** The long path to a successful *DPYD* testing implementation in the clinical practice of a National Cancer Center in Italy demonstrated that the development of pharmacogenetic guidelines and genotyping infrastructure standardization as well as capillary training and education activity for all the potential stakeholders are fundamental. However, only national health politics of test reimbursement and clear recommendations by drug regulatory agencies will definitely move the field forward.

## 1 Introduction

Despite the introduction of several innovative drugs in cancer treatment, fluoropyrimidines (fluorouracil and capecitabine) remain the backbone of systemic chemotherapies for a broad spectrum of solid tumors (Cavanna et al., 2006; Fernández-Martos et al., 2012; Bar-Ad et al., 2014; Heinemann et al., 2021). However, severe hematological and gastrointestinal toxicities occur in up to 30% of patients receiving fluoropyrimidines (van Kuilenburg et al., 2000; van Kuilenburg et al. 2010; van Kuilenburg, 2004; Amstutz et al., 2009; Meulendijks et al., 2015; Barin-Le Guellec et al., 2020; Sharma et al., 2021). The main fluoropyrimidines metabolizing enzyme is dihydropyrimidine dehydrogenase (DPD) representing the bottleneck in their detoxification pathway. Patients with decreased DPD activity are at risk of developing severe toxicity due to accumulation of fluoropyrimidines’ active metabolites. The presence of specific variants in the coding gene (*DPYD*) has been associated with DPD deficiency and is thus predictive of an increased risk of severe side effects (Terrazzino et al., 2013; Toffoli et al., 2015; Dalle Fratte et al., 2018; Henricks et al., 2018) and associated costs (Fragoulakis et al., 2019; Toffoli et al., 2019).

International authoritative consortia, including the Clinical Pharmacogenetics Implementation Consortium (CPIC) and the Dutch Pharmacogenetics Working Group (DPWG), have developed clinical pharmacogenetic (PGx) guidelines for fluoropyrimidines based on *DPYD* genotype in the clinical practice (Swen et al., 2011; Caudle et al., 2014; Amstutz et al., 2018; Bank et al., 2018; Lunenburg et al., 2020; Abdullah-Koolmees et al., 2021). In their most recent versions, both the CPIC and DPWG guidelines pointed out the importance of testing patients for the four genetic variants DPYD*2A (rs3918290), DPYD*13 (rs55886062), *DPYD* c.2846A>T (rs67373798), and *DPYD* c.1236G>A (rs56038477, tagging *DPYD-HapB3*) prior to treatment with fluoropyrimidines. In 2015, a joint committee of the Italian Society of Pharmacology (SIF) and the Italian Association of Medical Oncologists (AIOM) published the first version of their own PGx guidelines specifically addressing the gene-drug interaction of *DPYD* and fluoropyrimidines (SIF-AIOM, 2015; Gori et al., 2019).

Despite the guidelines availability, implementation in clinical practice has long been delayed due to many barriers, including the lack of appropriate genotyping and Information Technology (IT) platforms (Samwald et al., 2016), reimbursement issues, and low awareness of PGx among stakeholders (Just et al., 2017). Over the years, many initiatives have been undertaken to translate PGx results into the clinical practice. In this context, the European Union funded the Ubiquitous-Pharmacogenomics (U-PGx) study, which tested the implementation of PGx guidelines at 7 clinical sites in Europe within a prospective randomized clinical trial (PREemptive Pharmacogenomic testing for prevention of Adverse drug Reactions–PREPARE) (Manson et al., 2017; Swen et al., 2023). Our institute participated in the project as the only Italian implementation site, enrolling mainly oncology patients treated with fluoropyrimidines between 2017 and early 2020 (Cecchin et al., 2017; van der Wouden et al., 2017; Blagec et al., 2018; van der Wouden et al., 2020).

Driven by large prospective studies (Henricks et al., 2018), the attention of regulatory agencies on the predictive effect of DPD tests has increased over the years, prompting the European Medicines Agency (EMA) to publish recommendations in 2020 to improve appropriateness of fluoropyrimidine use (EMA, 2020). Later, in the same year, a similar recommendation was disseminated by the Italian Regulatory Agency (AIFA) to all Italian health centres (Italian Drug Agency, 2020).

The aim of this study is to describe how *DPYD* testing at the National Cancer Institute - Centro di Riferimento Oncologico (CRO) of Aviano has evolved over the last 10 years from a spontaneous research initiative to a structured diagnostic service. We describe how adopted PGx guidelines, genotyping technologies, and physicians’ awareness have changed over time. We also aimed to show how participation in the U-PGx implementation study and the publication of recommendations by European and Italian regulatory authorities have affected the *DPYD* diagnostic process in our center.

## 2 Materials and methods

The data analyzed in the present study were obtained from the internal database of the Experimental and Clinical Pharmacology of CRO -Aviano. It collects basic information on all the patients’ derived samples entering the pharmacogenetic diagnostics and is constantly updated by the staff involved in the diagnostic process. Eligible patients were inpatients and outpatients referred to Experimental and Clinical Pharmacology for *DPYD* testing between January 2012 and December 2022. In addition, data on the number of yearly fluoropyrimidine prescriptions were collected from the hospital pharmacy database to calculate the fraction of patients with a *DPYD* test prescription each year. Data collected included: demographic information (date of birth and sex), type of biological specimen (blood or saliva) and corresponding date of specimen collection, date of specimen receipt at the laboratory, materials stored for analysis (whole blood, buffy coat, plasma, or DNA), the reason for referral and genetic results generated for reporting.

Technical details of the adopted *DPYD* genotyping panels, as well as PGx guidelines and genotyping methods introduced over the years, were retrieved from laboratory registries to describe the gradual evolution of the *DPYD* testing service. Documents and correspondence with staff and physicians involved in the U-PGx project and the PREPARE protocol and associated standard operating procedures (SOP) were reviewed to describe the standardization process of laboratory procedures. In addition, documents and certificates related to ISO 15189 and external quality assessment were consulted for analysis. Based on the information collected, a descriptive data analysis was performed to outline the evolution of PGx diagnostic activity over the past decade.

## 3 Results

### 3.1 *DPYD* diagnostic service flow at experimental and clinical pharmacology CRO-Aviano over the years

Since 2012, the Experimental and Clinical Pharmacology Unit at CRO-Aviano has offered physicians genetic testing for *DPYD* polymorphisms in patients treated with fluoropyrimidines. Over the decade under consideration, this service has evolved considerably in line with the publication of literature evidence and corresponding PGx guidelines to include an increasing number of *DPYD* variants with an expected clinical impact.

Initially, *DPYD* testing was performed as part of the Institute’s translational pharmacogenetic research activities. Requests from prescribing oncologists were forwarded to the laboratory by telephone. After genotyping of the patient samples, the genetic results were directly reported to the requesting oncologist, who manually entered the results into the patient’s medical record.

After reimbursement for *DPYD* genetic analysis was approved at the regional level since 2014, the test was formally included in our center’s diagnostic services. The test prescription was electronically delivered to the laboratory by the prescribing physicians and an electronic diagnostic report was returned to the prescribing oncologist and stored in each patient’s electronic clinical folder. The *DPYD* diagnostic service was made available not only to CRO-Aviano patients but also to patients from other Institutes/Regions in Italy. Since 2020 and after publication of *DPYD* testing recommendation by AIFA, the *DPYD* genotyping prescription became widespread among physicians and reimbursed by the National Health System throughout the Italian territory. This also affected the number of prescriptions in our center.

### 3.2 *DPYD* variants and PGx guidelines over the years

**FIGURE 1:**
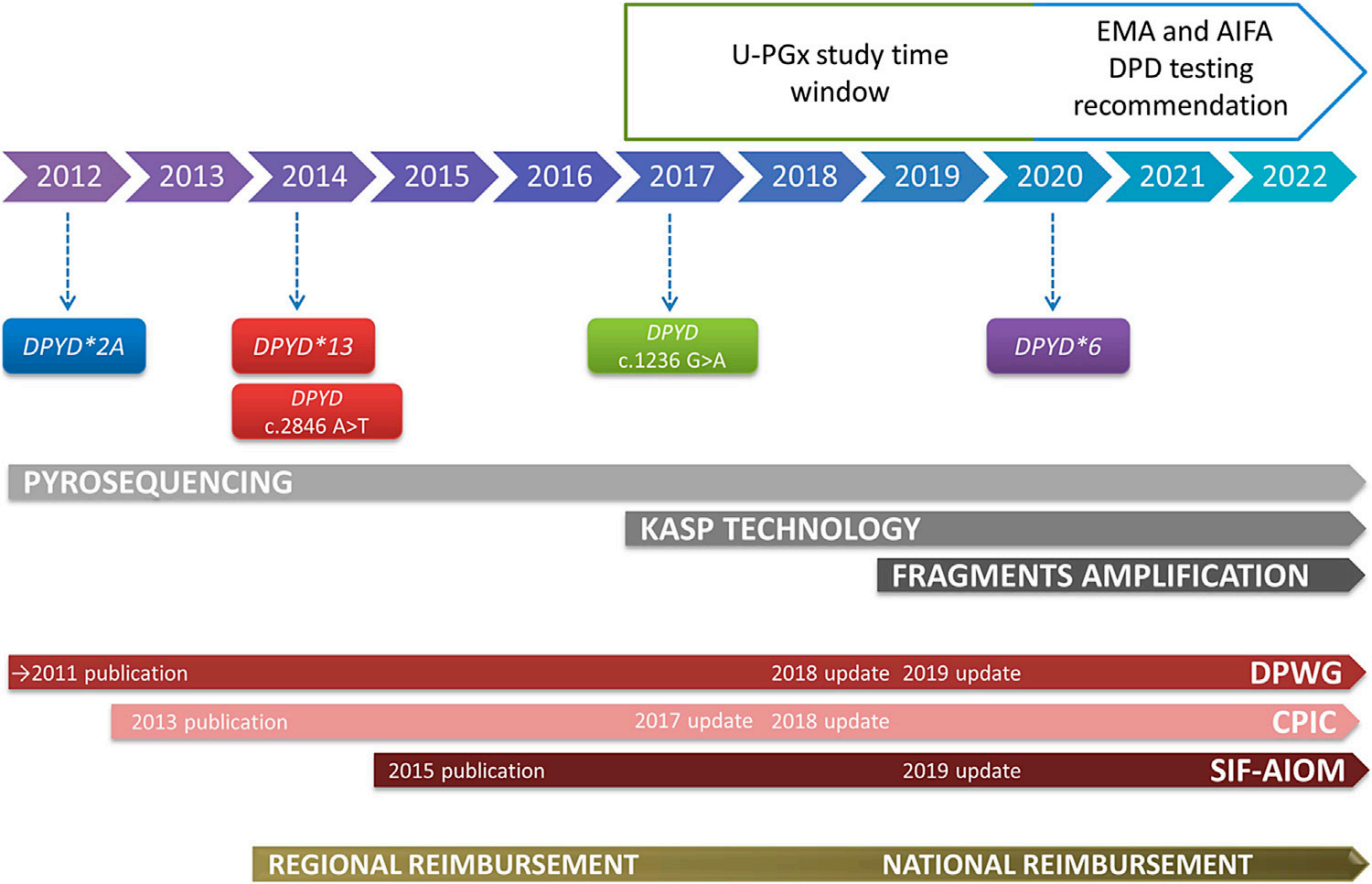
Timeline representing the evolution over the years of the *DPYD* panel tested, the genotyping technologies, the PGx guidelines adopted, and the test reimbursement at our center. The timeframe of participation to U-PGx project and publication of EMA and AIFA *DPYD* testing recommendation are highlighted. U-PGx, Ubiquitous Pharmacogenomics. EMA, European Medicines Agency. AIFA, Agenzia Italiana del Farmaco. DPD, DPYD, Di-hydroPYrimidine Dehydrogenase. DPWG, Dutch Pharmacogenetics Working Group. CPIC, Clinical Pharmacogenetics Implementation Consortium. SIF-AIOM, Società Italiana di Farmacologia- Associazione Italiana di Oncologia Medica. KASP, Kompetitive Allele Specific Polymerase chain reaction.

The *DPYD* genotyping panel and related recommendation have changed over the years (Figure 1).

In 2012, our laboratory started testing the DPYD*2A variant and adopted the 2011 DPWG guidelines of the Royal Dutch Pharmacists Association (Swen et al., 2011). The guideline recommended a 50% dose reduction in the presence of the DPYD*2A, variant allele or an alternative drug for carriers of two DPYD*2A variant alleles.

Since January 2014, DPYD*13 and *DPYD* c.2846A>T variants were added to the panel (Swen et al., 2011; Caudle et al., 2013). Accordingly, a 50% dose reduction was recommended for heterozygous carriers of DPYD*13 or *DPYD* c.2846A>T variant allele, as well as an alternative drug in the presence of two alleles among the two genetic polymorphisms considered.

In 2015, the collaboration between the Italian Association of Medical Oncology (AIOM) and the clinical Italian Society of Pharmacology (SIF) led to the publication of the Italian national recommendations for PGx analysis of *DPYD* in patients receiving fluoropyrimidines (SIF-AIOM, 2015), which were also considered in our laboratory as a reference for the *DPYD* diagnostic service. This first version of the Italian guidelines recommended *DPYD* testing for variants *2A, *13, and c.2846A>T regardless of a post-toxicity or pre-treatment approach. In particular, a 50% dose reduction was recommended for heterozygous carriers of any of these three variants, in line with international PGx guidelines (SIF-AIOM, 2015).

In 2017, we joined the European consortium U-PGx (www.upgx.eu) (van der Wouden et al., 2020; Swen, 2022) and participated in the clinical trial PREPARE (NCT03093818) a prospective, randomized European clinical trial aimed at evaluating the implementation of preemptive testing of a PGx panel, including *DPYD* for fluoropyrimidines (Swen et al., 2023). Based on the study protocol, we adopted the DPWG guidelines revised for the project purpose and made publicly available in 2018 (DPWG, 2018) for our diagnostic service. Accordingly, a fourth variant, *DPYD* 1236G>A (rs56038477, also known as tagging *DPYD-*HapB3) was added to the panel. In addition, the concept of the *DPYD* Gene Activity Score (GAS) was introduced for fluoropyrimidine dosing recommendations, consisting of a cumulative score (0–2) to assign a toxicity risk value to different combinations of *DPYD* genotypes (see Table 1). Consistent with the project objectives, several meetings were initially organized with prescribing physicians from different hospital departments to familiarize them with the study protocol and the potential of PGx in their practice and with this approach to drug prescribing.

**TABLE 1:** Comparison between 2018-updated and 2019-updated versions of DPWG guidelines based on *DPYD* Gene Activity Score (DPWG, 2018; DPWG, 2019; Lunenburg et al., 2020).

2018			2019		
**GAS**	**Diplotype**	**Recommendation**	**GAS**	**Diplotype**	**Recommendation**
**2**	*1/*1	Standard dose of FPs	**2**	*1/*1	Standard dose of FPs
**1.5**	*1/c.1236G>A, *1/c.2846A>T	Start with 75% of the standard dose or choose an alternative	**1.5**	*1/c.2846A>T *1/c.1236G>A	Start with 50% of the standard dose or avoid FPs
**1**	*1/*2A	Start with 50% of the standard dose or choose an alternative	**1**	*1/*2A	Start with 50% of the standard dose or avoid FPs
	*1/*13			*1/*13	
	c.2846A>T/c.2846A>T c.1236G>A/c.1236G>A		**PHENO**	c.2846A>T/c.2846A>T c.1236G>A/c.1236G>A	^a^Determine the residual DPD activity in mononuclear cells from peripheral blood and adjust the initial dose based on phenotype and genotype or avoid FPs
	c.2846A>T/c.1236G>A			c.2846A>T/c.1236G>A	
**0.5**	*2A/c.2846A>T	Start with 25% of the standard dose or choose an alternative		*2A/c.2846A>T *13/c.2846A>T *2A/c.1236G>A *13/c.1236G>A	
	*2A/c.1236G>A				
	*13/c.2846A>T				
	*13/c.1236G>A				
**0**	*2A/*2A	Choose an alternative. If an alternative is not possible: determine the residual DPD activity in mononuclear cells from peripheral blood and adjust the initial dose accordingly	**0**	*2A/*2A	Avoid fluorouracil and capecitabine or determine the residual DPD activity
	*13/*13			*13/*13	
	*2A/*13			*2A/*13	

PHENO: phenotyping; GAS: gene activity score; FPs: fluoropyrimidines.

^a^ DPD, enzyme activity cannot be predicted by genotype.

In November 2018, following the publication of a large prospective study testing the application of DPWG guidelines in the clinical practice (Henricks et al., 2018), CPIC published an update of their own guidelines (CPIC, 2018) suggesting that all carriers of a variant allele for one of the four variants, regardless the polymorphism, should receive a 50% dose reduction from the full standard starting dose. Accordingly, DPWG guidelines were also revised in August 2019 (DPWG, 2019; Lunenburg et al., 2020). Since 2019 we also adopted the DPWG guidelines revised version (Table 1) integrated with the Italian National guidelines SIF-AIOM (Gori et al., 2019).

According to the most updated version of the Italian National guidelines SIF-AIOM, published in October 2019, we introduced the test for DPYD*6 (*DPYD* 2194G>A, rs1801160) (Gori et al., 2019). This additional variant should only be tested in a post-toxicity setting if the patient experienced severe toxicity after starting fluoropyrimidine treatment. In the case of a heterozygous variant allele, a 15% dose reduction is recommended, increasing to 30% if the homozygous mutant allele (DPYD*6/*6) is present (Gori et al., 2019).

In the last 2019 version of DPWG guidelines a new category of GAS was introduced, called “PHENO,” which stands for “phenotyping”. In patients with the “PHENO” GAS, genetic testing for *DPYD* is in fact deemed not sufficient to determine the initial dose reduction, and measurement of residual enzymatic activity (phenotype) is suggested. Currently, our DPD testing service does not include phenotypic analysis of the enzyme.

On 30 April 2020, the publication of the EMA recommendations (EMA, 2020) on DPD testing represented a major driver for the implementation of pre-treatment PGx in our hospital and determined the general acceptance of *DPYD* testing before the administration of fluoropyrimidines. The European Directive was implemented at the national level by AIFA on 25 May 2020 (aifa.gov, 2020). This marked the final transition from a post-toxicity to a pre-treatment approach to *DPYD* testing requests from oncologists in Italy.

### 3.3 Evolution of the *DPYD* genotyping platform over the years

The genotyping technologies adopted by our laboratory have changed over the years (Figure 1). Since 2012, we have used pyrosequencing technology, a mini sequencing of a fragment containing the polymorphism of interest (PSQ48, Qiagen), to perform homemade tests for genetic variants of *DPYD*.

Since 2017, we have implemented a second technology based on end-point allele specific fluorescence detection. The method was implemented in the laboratory as part of the U-PGx study. As part of the patient journey in the study, a harmonized workflow was implemented to standardize laboratory practices and meet the requirements of the study protocol that pharmacogenetic results must be returned to prescribing physicians within three working days. The new workflow introduced in the U-PGx project was based on the use of the SNPline platform (LGC genomics, UK) using a Kompetitive Allele Specific Polymerase chain reaction (KASP) technology (van der Wouden et al., 2020).

A third method based on allele specific fragment amplification is available in the laboratory and has been used for the four polymorphisms in *DPYD* since December 2019.

Our diagnostic workflow includes independent validation of results using any two of these available methods. Concerning the analysis turnaround time, it is related to the optimization of the entire workflow, which includes blood collection and processing, reception of the analysis, DNA extraction, double genotyping procedure (using two independent methods), data analysis, and preparation of the clinical report. We noted that the use of any two of the three available methods does not affect the turnaround time of the entire process, which is now set at 3 days. Considering that samples are pooled and analyzed once a week, the maximum turnaround time is 1 week.

Since 2020, our laboratory has been undergoing an accreditation program in accordance with the International Standard ISO 15189 (“Medical laboratories–Requirements for quality and competence”), which is specifically tailored to the activities of medical laboratories and covers both the requirements for the quality system and the competence of laboratory personnel. Since 2019, the laboratory also participates in the External Quality Assessment (EQA) for laboratories delivering pharmacogenetic diagnostic tests offered by the European Molecular Genetics Quality Network (EMQN) (emqn.org).

### 3.4 IT genetic data management

Since 2014, *DPYD* test prescriptions and related reports have been incorporated into the existing Regional digitalized molecular diagnostic tests prescribing and reporting system. Once the oncologist prescribes a *DPYD* test in the hospital management system, blood/saliva sample collection labels are automatically generated with a unique code to track the sample sent to the laboratory.

After analysis, a genetic report is generated via the laboratory system IT DNLAB^®^ indicating the type of biological material from which the DNA was extracted, the method used for genetic analysis, the genetic results of the *DPYD* variants analyzed, and the appropriate dosing recommendations. The report is technically and clinically validated, digitally signed, and stored as a. pdf file in the patient’s electronic health record. This approach has been limited by the lack of an interactive clinical decision support system (CDSS) that could improve the application of PGx guidelines in clinical practice.

To bridge the gap, the FARMAPRICE project was launched in 2017, funded by POR FESR 2014-2020, to develop a prototype CDSS to help physicians manage their patients’ genetic data and translate them into precise prescribing indications (Roncato et al., 2019). This prototype will help physicians make safe and appropriate prescriptions. When a drug to be prescribed is entered into the FARMAPRICE platform, the physician assesses the presence of specific and validated gene-drug interactions that highlight the presence of a potentially actionable genotype for the patient by matching the PGx guideline repository with the genetic repository. If an actionable genotype is found, the physician receives a PGx-based recommendation with the appropriate level of evidence. The FARMAPRICE prototype is a ready-to-use platform that can be integrated into the hospital management system. However, implementation has been delayed due to the outbreak of COVID-19 and is pending at the time of writing.

The participation in the PREPARE study allowed us to use new IT solutions for genetic data reporting, including the use of a Genetic Information Management Platform (GIMS). GIMS provided standardized diagnostic reports including detailed genetic recommendations based on DPWG guidelines, which were constantly updated (Blagec et al., 2022). In addition, the U-PGx project provided patients with a Safety Code Card (SCC) that digitally contained their pharmacogenetic profile. The SCC was a user-friendly tool that allowed the report to be accessed in detail and in digital form via the QR code scan, so that patients and healthcare professionals could access it at any time via a smartphone.

### 3.5 Diagnostic activity trend over the years

During the reference period, 1,987 *DPYD* test requests were referred to the Experimental and Clinical Pharmacology Unit at National Cancer Institute CRO-Aviano. Out of the 1,987 patients, 974 (49.1%) were female and 1,013 (50.9%) were male, with mean age of 64.8 and 65.1 years, respectively. Almost 95% (1,885 samples) were inpatients, while 5.2% (102 samples) were patients from outside the hospital. Most of the collected samples were blood samples (1,855; 93.3%) and only 6.7% (132 samples) were saliva samples.

**FIGURE 2:**
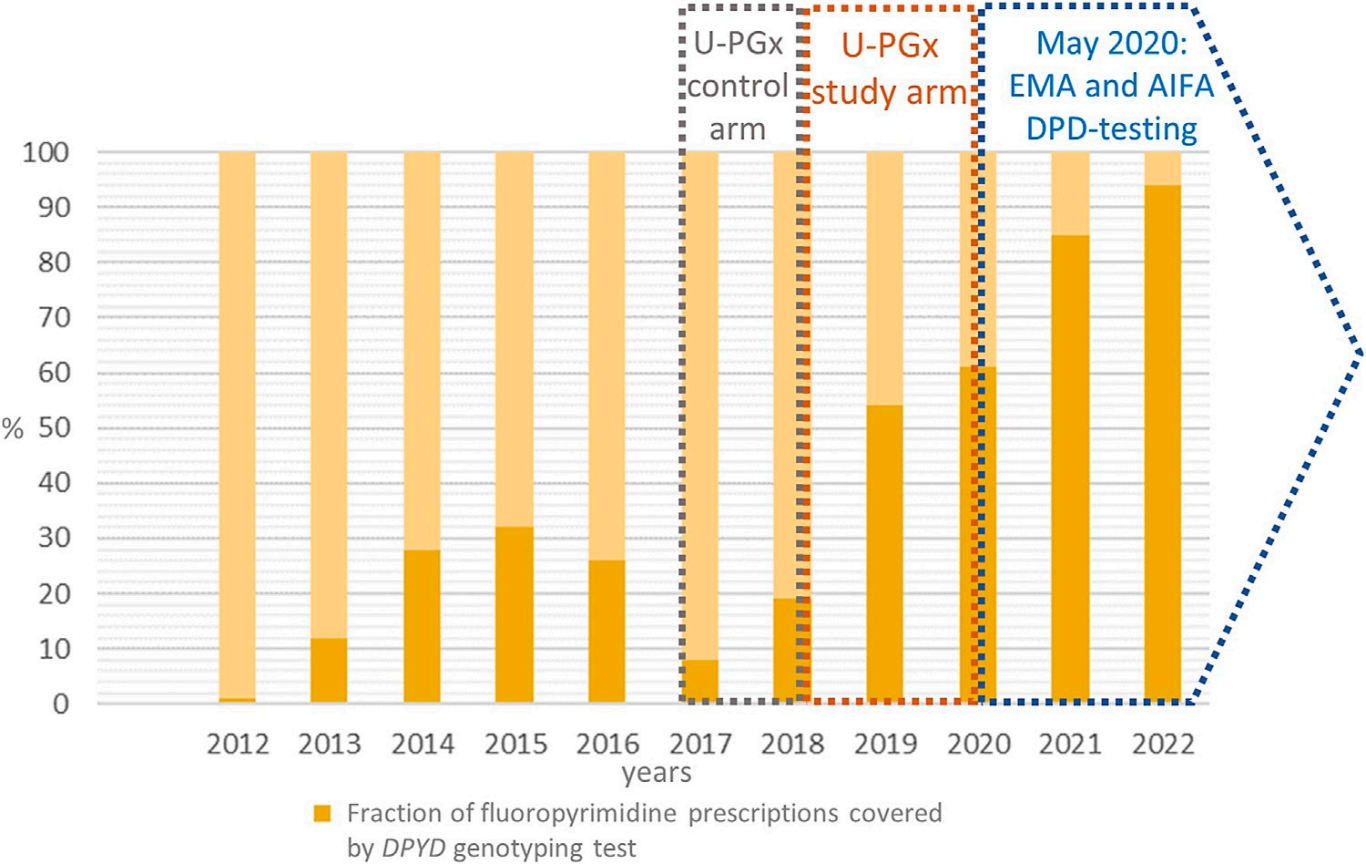
The figure reports the fraction (in percentage) of the fluoropyrimidines prescription at our center that were associated to a *DPYD* test prescription over the years. The timeframe of participation to U-PGx project (control and study arm) as well as the publication of EMA and AIFA *DPYD* testing recommendation are highlighted. U-PGx, Ubiquitous Pharmacogenomics. EMA, European Medicines Agency. AIFA, Agenzia Italiana del FArmaco. DPD, DPYD, Di-hydroPYrimidine Dehydrogenase

The number of inpatients receiving a fluoropyrimidine prescription each year was retrieved from the hospital pharmacy (patients who were already tested for *DPYD* variants were excluded from the count) and was compared to the number of *DPYD* tests delivered for inpatients each year. As reported in Table 2 and Figure 2 the percentage of tested inpatients increased over the years reaching 94% in 2022.

**TABLE 2:** Yearly trend of *DPYD* genotyping prescriptions referred to the Experimental and Clinical Pharmacology Unit at National Cancer Institute CRO-Aviano and the fraction of fluoropyrimidines prescriptions covered by the test.

Year	Total *DPYD* test (n)	Internal FPs prescriptions (n)	Inpatients *DPYD* test (n)	Inpatients *DPYD* test coverage (%)	Pre-treatment *DPYD* genotyping requests	
					n	%
2012	10	500	7	1	9	90
2013	49	414	49	12	49	100
2014^a^	114	408	114	28	113	99.1
2015	131	402	127	32	115	87.8
2016	111	399	102	26	95	85.6
2017	37	405	33	8	30	81
2018	94	474	88	19	89	94.7
2019	299	534	287	54	295	98.6
2020^b^	299	479	290	61	297	99.3
2021	436	463	395	85	436	100
2022	407	420	393	94	407	100

^a^ Introduction of regional reimbursement.

^b^ Nationwide coverage of test reimbursement. Pts: patients; FPs: fluoropyrimidines.

The number of patients referred for post-toxicity testing totaled 52 (2.6%), whereas the number of samples referred for pre-treatment genotyping was 1,935 (97.4%). A progressive increase in the rate of pre-treatment versus post-toxicity testing was observed over the years (Table 2).

Table 2 highlights also the trend of patients’ inclusion in the *DPYD* diagnostic program in our center between January 2012 and December 2022. In the 2017-2018 time window, the number of yearly requests remained stable or slightly decreasing, due to the center’s participation in the standard-of-care arm of the PREPARE clinical trial. After the switch to the PREPARE study arm in October 2018 (Swen et al., 2023) and the publication of the DPD test recommendation by EMA and AIFA in May 2020, the number of test requests increased dramatically until the last year of observation (2022).

## 4 Discussion

Awareness of the clinical value of *DPYD* testing to limit the risk of severe toxicity to fluoropyrimidines has notably increased over the past decade (Deenen et al., 2011; Meulendijks et al., 2015; Lunenburg et al., 2016; Dalle Fratte et al., 2018). We report here the experience of a tertiary-level hospital in Italy (National Cancer Institute, CRO-Aviano) with the implementation of *DPYD* genetic polymorphism testing in patients since 2012.

Overall, as with other PGx testing, the adoption of *DPYD* testing in hospitals has been hampered by several previously discussed barriers, such as the need for common national and international pharmacogenetic guidelines, reliable genotyping technology with acceptable turnaround time, and IT technologies suitable for managing genetic data as part of standard clinical workflow (Swen et al., 2011; Amstutz et al., 2018; Martens et al., 2019; Lunenburg et al., 2020; Begré et al., 2022).

Prescribers awareness of the clinical relevance of the tests is considered another relevant barrier to upfront *DPYD* testing in the clinical practice (Formea et al., 2013; Haga et al., 2015; Just et al., 2017, 2019; Giri et al., 2018). The herein reported data show that over the years, not only has the absolute number of tests prescribed increased, but so has the trend from a post-toxicity to a pre-treatment approach, attesting the increasing awareness among oncologists of the importance of adverse drug reactions from *DPYD* genotyping. This could be also related to the active involvement of oncologists in prospective clinical trials such as the U-PGx project (Swen et al., 2023).

The management of genetic data in a clinical context could be another barrier to straightforward implementation of PGx testing in clinical practice (Khelifi et al., 2017). Our diagnostic reporting service has evolved from a paper report delivered only to the prescribing physician, to an electronic report that is included in the patient’s health repository and available to any physician with access to the patient’s health data (Roncato et al., 2019; Qin et al., 2022). However, we recognize that a clinical decision support system in which the patient’s genetic data interact with the medication prescribing system would be the best way to facilitate the integration of genotyping results into the clinical workflow. With this in mind, the CDSS prototype FARMAPRICE was developed with the aim of integrating genetic data into the digital medical record of patients from the CRO-Aviano (Roncato et al., 2019), although no results on clinician acceptance of the tool are currently available. Another approach, within the U-PGx project, was the introduction of the Safety Code Card, a wearable CDSS provided in the patient’s hand. However, the latter was hardly adopted by Italian patients in the project, probably due to the high average age of cancer patients, which may affect the ability to use the technologies, or, more simply, to the lack of new drugs prescription given the high mortality rate of the disease (Blagec et al., 2022).

Over the reference time, the number of *DPYD* variants analyzed and the laboratory methods have also changed according to the continuous evolution of the scientific literature and the pharmacogenetic guidelines (Swen et al., 2011; Amstutz et al., 2018; Lunenburg et al., 2020; Abdullah-Koolmees et al., 2021). In the most recent years several European countries developed their own *DPYD* testing panels (Martens et al., 2019; Wörmann et al., 2020; Begré et al., 2022) adding in some cases specific *DPYD* variants in addition to the four variants panel (García-Alfonso et al., 2022). In Italy, a joint committee promoted by SIF-AIOM has developed specific Italian PGx guidelines for *DPYD* testing since 2015, and an updated version was made available in 2019 (SIF-AIOM, 2015; Gori et al., 2019). The *DPYD* pretreatment panel recommended in the SIF-AIOM guidelines is in line with the recommendations of the CPIC and DPWG international consortia. In Italy, an additional *DPYD* variant (DPYD*6) is recommended for testing in case of severe toxicity, based on the results of some pharmacogenetic association studies reporting a higher risk of toxicity in carriers of this polymorphism (Boige et al., 2016; Ruzzo et al., 2017; Henricks et al., 2018).

The Italian guidelines do not include recommendations for DPD phenotyping by assessing residual DPD enzyme activity from peripheral blood by analysis of uracil (U) and dihydrouracil (UH_2_) metabolite plasma concentrations (Van Kuilenburg et al., 1999; Pallet et al., 2020; Ockeloen et al., 2021). Although phenotyping by UH_2_/U in peripheral blood mononuclear cells is a direct measure of DPD activity and could reveal a greater number of patients at risk for toxicity, regardless of genetic profile, its application is hampered by several technical limitations. The lack of standardization in the timing of blood collection and processing protocols may influence results, and makes it difficult to directly correlate this ratio with the enzyme activity (de With et al., 2022). Although this is a valuable approach whose effectiveness is demonstrated by its acceptance in other countries such as France (Laures et al., 2022), a DPD phenotyping service is poorly provided by Italian public laboratories.

The lack of clear reimbursement strategies remains a critical barrier to the implementation of pharmacogenetic testing in practice worldwide, in some cases limiting the use of *DPYD* testing to funded projects only (Faulkner et al., 2012; Luzum et al., 2017). Many health economic issues are autonomously managed by different Italian regions. In our case, this led to inhomogeneity in the possibility of having the test reimbursed on the Italian territory. In the Friuli Venezia Giulia region, where our center is based, the pharmacogenetic test has been reimbursed since 2014. This was the first event that improved the uptake of the test by clinicians, as the number of patients referred to the *DPYD* genotyping service doubled between 2013 and 2014. The *DPYD* analysis service at the National Cancer Institute CRO-Aviano was made available to patients referred to the hospital as well as to patients from outside hospital at the regional and national level and become a benchmark for several national centers.

However, the crucial step that led to the inclusion of *DPYD* testing in the clinical practice of our center was the introduction of specific recommendations for DPD testing before fluoropyrimidines prescription by the European (EMA) and Italian (AIFA) regulatory authorities (EMA, 2020; aifa.gov, 2020). Since 2020, pre-treatment *DPYD* testing has been reimbursed in Italy. As our results show, the number of patients tested for *DPYD* before treatment almost doubled between 2020 and 2021 to reach a stable plateau of almost 400 inpatients per year, which is more than 90% of the average number of patients prescribed a fluoropyrimidine in our center in 2022.

Although our results are based on a unique observation point in Italy, where early adoption of testing was driven by specific local health policies and participation in important international pharmacogenetic projects, we observed that similar trends were reported in other European contexts. In recent years, some examples of the introduction of *DPYD* testing into the clinical practice with the support of local health authorities have been reported (Martens et al., 2019; Wörmann et al., 2020; Begré et al., 2022; García-Alfonso et al., 2022). Recently, a large survey was conducted in several European countries, including Italy, providing an overview of the status of DPD testing implementation in Europe and how this was affected by the publication of the EMA recommendation in 2020. As in the herein presented results, the EMA recommendation was the key event affecting the number of test prescriptions and the revision of national reimbursement guidelines, stimulating the publication of national guidelines in most European countries (de With et al., 2023).

## 5 Conclusion

*DPYD* testing is widely recognized as an important strategy to increase fluoropyrimidines treatment safety, however, its implementation in clinical practice is still struggling to become part of routine testing in some parts of the world (Baker et al., 2023). The example of the implementation pathway in our center in Italy shows once again that the success of this process depends on several factors, including disclosure of the value of *DPYD* testing among stakeholders, standardization of laboratory workflows, and adoption of straightforward IT technology. However, the final and critical step for implementing the test into routine practice is the availability of a clear regulatory recommendation by drug regulatory authorities and the establishment of a reimbursement policy.

## Data Availability

The raw data supporting the conclusion of this article will be made available by the authors, without undue reservation.
